# First description of clinical presentation of piscine orthoreovirus (PRV) infections in salmonid aquaculture in Chile and identification of a second genotype (Genotype II) of PRV

**DOI:** 10.1186/s12985-016-0554-y

**Published:** 2016-06-13

**Authors:** Marcos G. Godoy, Molly J. T. Kibenge, Yingwei Wang, Rudy Suarez, Camila Leiva, Francisco Vallejos, Frederick S. B. Kibenge

**Affiliations:** Centro de Investigaciones Biológicas Aplicadas (CIBA), Diego de Almagro Norte 1013, No. 8, Puerto Montt, Chile; Facultad de Ciencias, Universidad San Sebastián, Lago Panguipulli 1390, Puerto Montt, Chile; ETECMA, Diego de Almagro Norte 1013, No. 10, Puerto Montt, Chile; Doctorado en Acuicultura, Programa Cooperativo Universidad de Chile, Universidad Católica del Norte, Antofagasta, Chile; Doctorado en Acuicultura, Programa Cooperativo Universidad de Chile, Pontificia Universidad Católica de Valparaíso, Valparaíso, Chile; Department of Pathology and Microbiology, Atlantic Veterinary College, University of Prince Edward Island, 550 University Ave, Charlottetown, PEI C1A 4P3 Canada; School of Mathematical and Computational Sciences, University of Prince Edward Island, 550 University Ave, Charlottetown, PEI C1A 4P3 Canada; Friosur, Aysen, Chile; Present Address: Aquagestión S.A. Panamericana Sur 428, Puerto Montt, Chile

**Keywords:** Heart and skeletal muscle inflammation, HSMI, HSMI-like, Piscine orthoreovirus, PRV, Atlantic salmon, coho salmon

## Abstract

**Background:**

Heart and skeletal muscle inflammation (HSMI) is an emerging disease of marine-farmed Atlantic salmon *Salmo salar*, first recognized in 1999 in Norway, and recently associated with piscine orthoreovirus (PRV) infection. To date, HSMI lesions with presence of PRV have only been described in marine-farmed Atlantic salmon in Norway. A new HSMI-like disease in rainbow trout *Oncorhynchus mykiss* associated with a PRV-related virus has also been reported in Norway.

**Methods:**

Sampling of Atlantic salmon and coho salmon was done during potential disease outbreaks, targeting lethargic/moribund fish. Fish were necropsied and tissues were taken for histopathologic analysis and testing for PRV by RT-qPCR assay for segment L1 and conventional RT-PCR for PRV segment S1. The PCR products were sequenced and their relationship to PRV strains in GenBank was determined using phylogenetic analysis and nucleotide and amino acid homology comparisons.

**Results:**

The Atlantic salmon manifested the classical presentation of HSMI with high PRV virus loads (low Ct values) as described in Norway. The coho salmon with low Ct values had myocarditis but only in the spongy layer, the myositis of red muscle in general was mild, and the hepatic necrosis was severe. Upon phylogenetic analysis of PRV segment S1 sequences, all the Chilean PRV strains from Atlantic salmon grouped as sub-genotype Ib, whereas the Chilean PRV strains from coho salmon were more diversified, grouping in both sub-genotypes Ia and Ib and others forming a distinct new phylogenetic cluster, designated Genotype II that included the Norwegian PRV-related virus.

**Conclusions:**

To our knowledge the present work constitutes the first published report of HSMI lesions with presence of PRV in farmed Atlantic salmon outside of Europe, and the first report of HSMI-like lesions with presence of PRV in coho salmon in Chile. The Chilean PRV strains from coho salmon are more genetically diversified than those from Atlantic salmon, and some form a distinct new phylogenetic cluster, designated Genotype II.

**Electronic supplementary material:**

The online version of this article (doi:10.1186/s12985-016-0554-y) contains supplementary material, which is available to authorized users.

## Background

The main salmon species farmed in Chile include Atlantic salmon *Salmo salar*, coho salmon *Oncorhynchus kisutch*, and rainbow trout *O. mykiss*. The appearance of important diseases posing a risk to the salmon industry in Chile has been related directly to the increase in production [1].

In 2010 a new virus called piscine orthoreovirus (PRV) was described [2] as belonging to the family *Reoviridae*, subfamily *Spinareovirinae*, and has been proposed as a member of the genus *Orthoreovirus* [3]. PRV is an aquatic reovirus that is ubiquitous in Atlantic salmon in Norwegian aquaculture [4, 5], and has also been detected in wild Atlantic salmon [2] and sea-trout *S. trutta* [5, 6] and in certain marine fish species (Atlantic herring *Clupea harengus*, Capelin *Mallotus villosus*, Atlantic horse mackerel *Trachurus trachurus*, and Great silver smelt *Argentina silus*) along the coast of Norway [7]. Outside of Norway, PRV has been detected by RT-qPCR in farmed Atlantic salmon in Chile [8] and Ireland [9], in wild and farmed Atlantic salmon in Denmark [10], in farmed Atlantic salmon and wild chum salmon *O. keta*, rainbow trout *O. mykiss* and cutthrout trout *O. clarkii* in British Columbia-Canada [8], in marine-farmed Chinook salmon *O. tshawytscha* [11], in wild coho salmon *O. kisutch* from Alaska-USA [12], and recently in hatchery Chinook salmon and coho salmon in Washington state-USA [13]. In fact PRV was reported as being enzootic in farmed and wild salmonids on the Canada/US Pacific Coast [14].

Piscine orthoreovirus is associated with heart and skeletal muscle inflammation (HSMI), a disease of marine-farmed Atlantic salmon first reported in Norway and Scotland. HSMI affects fish 4 to 8 months after transfer to sea during the fattening stage, and is accompanied by low to moderate mortality (~20 %) but morbidity rates have been reported as high as 100 % [15, 16]. Confirmatory diagnosis of the disease is by histopathological examination and the classical presentation is epicarditis, endocarditis, myocarditis, and myositis and necrosis of the red skeletal muscle [15–17]. Outside of Europe, integrated technologies were used to diagnose a potential HSMI in farmed Atlantic salmon samples collected from an aquaculture facility in 2013–2014 in British Columbia-Canada [18]. In Chile, a technical preliminary report on PRV in farmed Atlantic salmon found no associated clinical signs and only mild nonspecific histopathological lesions resembling HSMI [19]. The virus was found in 64 % of the Atlantic salmon farms analyzed, and of 700 fish tested, 323 were positive for PRV, representing a prevalence of 46 % [20].

There is a clear need for histopathological analysis of appropriate (i.e., “fit for purpose”) tissue samples for confirmatory diagnosis of HSMI especially since PRV can be present asymptomatically in a wide range of fish species [5–8, 12, 21, 22]. Most recently, a new disease in rainbow trout similar to HSMI and associated with a PRV-related virus was also reported in Norway [23]. The disease was observed starting in fall 2013, in three freshwater hatcheries and up to 4 months after sea water transfer in two sea farms on the west coast of Norway [23]. A viral 561-bp nucleotide sequence obtained from these cases (GenBank Accession number LN680851) had 85 % identity to segment S1 of the PRV type strain Salmo/GP-2011/NOR (GenBank Accession number GU994022). No controlled laboratory studies have been done to link the PRV-related virus to the pathological findings.

In this study, we describe for the first time, the clinical presentation of PRV infections in two of the three major farmed salmonid species in Chile. We provide the first description of HSMI in Atlantic salmon outside Europe and the first detection of HSMI-like disease in coho salmon. The histopathological findings in the coho salmon are novel and appear to be associated with the presence of PRV. We also undertook further genetic characterization of PRV isolates in this study and including PRV S1 sequences available in GenBank [24] in order to improve on the information about the genetic diversity of PRV.

## Results and discussion

### Clinical presentation and gross pathology of HSMI cases in Atlantic salmon and HSMI-like cases in coho salmon

In the Atlantic salmon the affected fish had reduced feed intake, were lethargic, and some swum close to the net pen and in the direction of the water current. An additional movie file shows this in more detail (see Additional file 1). Some of the moribund fish lost equilibrium and either recovered the equilibrium or died. In the coho salmon the affected fish had reduced feed intake, which was followed by appearance of dead fish floating in the surface water mainly in the corner of the net pen.

**Fig. 1:**
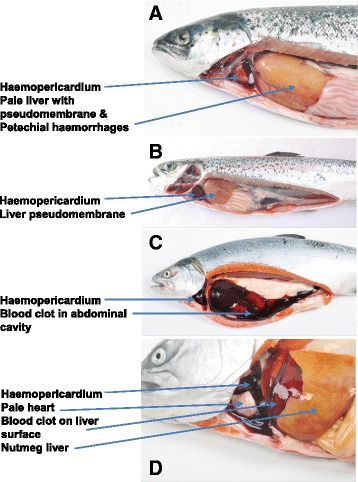
Main gross pathology findings in Chilean farmed Atlantic salmon *Salmo salar* and coho salmon *Oncorhynchus kisutch*: Panels **a** and **b**: Atlantic salmon with characteristic gross lesions of heart and skeletal muscle inflammation (HSMI). Panels **c** and **d**: Coho salmon with gross lesions of HSMI-like disease

63 Atlantic salmon were analyzed from two farm sites, which were selected from 17 Atlantic salmon farm sites studied, and 85 coho salmon were analyzed from two farm sites, which were selected from nine coho salmon farm sites studied. The farm sites were chosen in order to increase the probability of detecting HSMI disease in Atlantic salmon or HSMI-like disease in coho salmon with the goal to establish a causal relationship between PRV and HSMI. Thus targeted sampling was carried out by an experienced Veterinarian at peak mortality during a suspected outbreak of HSMI or HSMI-like clinical disease in the absence of any pathogen other than PRV, and the samples for histopathological examination were taken from selected fish showing HSMI or HSMI-like gross pathology. All the samples were collected from 2012 to 2015. Table 1 summarizes the frequency of the significant gross pathology findings noted from Atlantic salmon affected with HSMI and coho salmon affected with HSMI-like disease. In Atlantic salmon the most common lesions related to HSMI were pale and yellow liver in 60.3 %, clotted blood in cardiac cavity (haemopericardium) in 28.6 %, and pale gill and pale heart, each, in 19 % of the fish necropsied (Table 1). The representative gross pathology findings in Atlantic salmon affected with HSMI are shown in Fig. 1a and b. In coho salmon the most common lesions related to HSMI-like disease were yellow liver and haemopericardium in 55.3 %, pale heart in 43.5 %, biliary cholestasis in 31.8 %, ascites in 20.0 % and clotted blood in the abdominal cavity in 17.6 % of the fish necropsied. Unlike the Atlantic salmon, some of the coho salmon also had spinal fracture (22.4 %) and kidney rupture (11.8 %) (Table 1). The representative gross pathology findings in coho salmon affected with HSMI-like disease are shown in Fig. 1c and d.

**Table 1:** Gross pathology findings of Atlantic salmon affected with heart and skeletal muscle inflammation (HSMI) and coho salmon affected with HSMI-like disease

Gross pathology findings	Frequency^1^ (%)	
	Atlantic salmon	Coho salmon
Yellow liver	60.3 %	55.3 %
Hemopericardium	28.6 %	55.3 %
Pale gill	19.0 %	56.5 %
Nutmeg liver	19.0 %	-
Pale heart	19.0 %	43.5 %
Splenomegally	17.5 %	29.4 %
Visceral melanosis	15.9 %	-
Petechiae in visceral fat	15.9 %	7.1 %
Liver pseudomembrane	12.7 %	-
Ascites	12.7 %	20.0 %
Congestion in intestines	11.1 %	-
Hemorrhagic eye	11.1 %	-
Hepatomegally	9.5 %	-
Renomegally	4.8 %	10.6 %
Fluid content in stomach	3.2 %	14.1 %
Congestion in pyloric caeca	3.2 %	-
Clotted blood in the abdominal cavity	3.2 %	17.6 %
Jaundice	1.6 %	24.7 %
Biliary cholestasis	-	31.8 %
Spinal fracture	-	22.4 %
Kidney rupture	-	11.8 %
Lipid cysts in visceral fat	-	7.1 %
Accumulation of feed oil in the stomach	-	5.9 %
Exophthalmia	-	2.4 %
Hydropericardium	-	2.4 %
Gastric dilatation (bloat)	-	2.4 %

^1^Frequency expressed as a percentage of fish with lesion over total examined. 63 Atlantic salmon were necropsied from two farms corresponding to four sampling times; 85 coho salmon were necropsied from two farms corresponding to four sampling times

The clinical presentations described in Atlantic salmon and coho salmon are consistent with a circulatory disturbance similar to that described by Kongtorp et al. [15, 17] and Ferguson et al. [16] for Atlantic salmon affected with HSMI, and by Olsen et al. [23] for rainbow trout affected with HSMI-like disease. In the case of coho salmon, we additionally observed spontaneous spinal fracture and kidney rupture. These lesions were observed in coho salmon that had fast growth, with good body condition and abundant visceral fat, and they have been linked to a non-infectious condition of metabolic type. A gross lesion that was more common in coho salmon was jaundice; this had been previously related to Jaundice coho salmon syndrome, a condition previously associated with infectious salmon anaemia virus [25], however all samples in the present study were negative in the RT-qPCR assay for ISAV. Another disease in Chilean coho salmon related with jaundice is infectious haemolytic anaemia [26]. The main histopathological findings of this disease are haemorrhages in all organs and severe haemosiderosis accompanied by erythrophagocytosis in the kidney and spleen. However, none of the coho salmon cases analyzed in this study had haemosiderosis although erythrophagocytosis was seen in the spleen (see Table 2 below). Jaundice was recently described by Garver et al. [11] in Chinook salmon positive for PRV, but the condition could not be reproduced experimentally although it was possible to transmit the virus to the experimental fish.

**Table 2:** Nature and frequency^1^ of significant histopathology lesions in tissues of Atlantic salmon affected with heart and skeletal muscle inflammation (HSMI) and coho salmon affected with HSMI-like disease

Tissue			Atlantic salmon			Coho salmon		
			Histopathological description	Diagnosis	Frequency	Histopathological description	Diagnosis	Frequency
Heart	Epicardium		Multifocal to diffuse infiltration of mononuclear cells.	Mononuclear epicarditis, subacute, multifocal to diffuse, moderate to severe.	33/36	Multifocal to diffuse infiltration of mononuclear cells.	Mononuclear epicarditis, subacute, multifocal to diffuse, moderate to severe.	18/18
	Myocardium	Spongy layer	Multifocal to diffuse infiltration of mononuclear cells of spongy layer and compact layer. Multifocal to diffuse myocardial degeneration & necrosis.	Mononuclear myocarditis, degenerative, necrotic, subacute to chronic, multifocal to diffuse, moderate to severe.	33/36	Multifocal to diffuse infiltration of mononuclear cells of spongy layer. Some mononuclear cells are present in the compact layer. Focal to multifocal myocardial degeneration & necrosis.	Mononuclear myocarditis, degenerative, necrotic, subacute to chronic, multifocal to diffuse, moderate to severe.	15/18
		Compact layer				Not significant findings		
Red muscle			Multifocal to diffuse infiltration of mononuclear cells in red muscle. Multifocal to diffuse myocytic degeneration & necrosis.	Mononuclear myocarditis, degenerative, necrotic, subacute to chronic, Multifocal to diffuse, moderate to severe.	30/36	Multifocal to diffuse infiltration of mononuclear cells in red muscle.	Mononuclear myocarditis, Multifocal to diffuse, mild to moderate.	12/18
Liver			Blood vessels surrounded by a mononuclear cell infiltrate. Degeneration, necrotic, hemorrhagic, acute to subacute, focal to multifocal, moderate to severe.	Mononuclear hepatitis, necrotic, hemorrhagic, acute to subacute, focal to multifocal, moderate to severe.	16/36	Blood vessels surrounded by a mononuclear cell infiltrate. Degeneration, necrotic, acute to subacute, focal to multifocal, mild to moderate.	Mononuclear perivascular hepatitis, necrotic, acute to subacute, focal to multifocal, mild to moderate.	14/18
Tissue	Atlantic salmon				Coho salmon			
	Histopathological description		Diagnosis	Frequency	Histopathological description		Diagnosis	Frequency
Spleen	Diffuse infiltration of mononuclear cells, interstitial. Diffuse congestion with evidence of increase in circulating granulocytes & intravascular erythrophagia.		Splenitis, subacute, diffuse, moderate.	11/36	Diffuse infiltration of mononuclear cells, interstitial. Diffuse congestion with evidence of increase in circulating granulocytes & intravascular erythrophagia.		Splenitis, subacute, diffuse, moderate.	10/18
Kidney	Diffuse infiltration of mononuclear cells, interstitial.		Interstitial nephritis, subacute, diffuse, moderate.	4/36	Diffuse infiltration of mononuclear cells, interstitial.		Interstitial nephritis, subacute, diffuse, moderate.	11/18
Pyloric caeca	Extensive proliferation of fibroblasts-like cells, mononuclear, eosinophilic cells with eccentric nucleus & macrophage-like cells. Large empty vacuoles adjacent to the pancreatic area observed in some cases.		Vaccine induced peritonitis	11/36	No significant findings.		No significant findings.	18/18
Gill	Lamellar fusion across multiple filaments.		Branchiosis multifocal, moderate.	3/36	Lamellar fusion across multiple filaments. Numerous interlamellar vesicle formation. *Neoparamoeba perurans* showing nucleus & “parasome". Granular eosinophilic intracytoplasmic inclusions in hypertrophied epithelial cells.		Focal to multifocal bacterial & parasitic hyperplasia, subacute to chronic, moderate to severe, with *Paramoeba perurans* & epitheliocystis-like inclusions.	16/18
Posterior gut	Multifocal to diffuse infiltration of mononuclear cells in lamina propria & sub mucosa.		Mononuclear enteritis, multifocal to diffuse, moderate.	5/36	Multifocal to diffuse infiltration of mononuclear cells in lamina propria & sub mucosa.		Mononuclear enteritis, multifocal to diffuse, moderate.	4/18

^1^Frequency corresponds to number of fish with lesion over total examined. 36 Atlantic salmon were examined from two farms corresponding to four sampling times; 18 coho salmon were examined from two farms corresponding to four sampling times

### Histopathology of HSMI cases in Atlantic salmon and HSMI-like cases in coho salmon

**Fig. 2:**
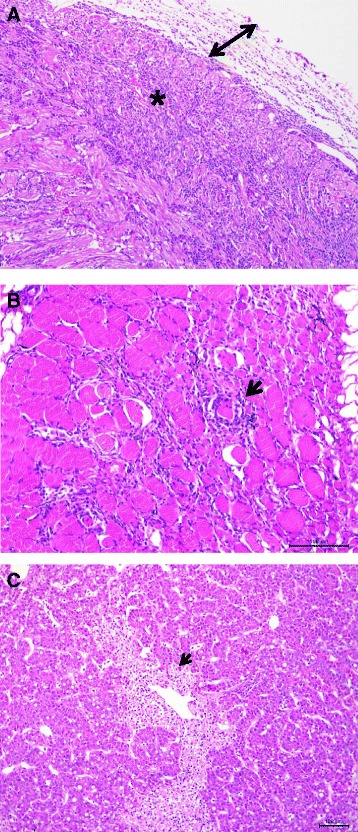
Photomicrographs showing the main microscopic findings in farmed Chilean Atlantic salmon *Salmo salar* with HSMI-like disease. Panel **a**: Heart section stained with haematoxylin and eosin (H&E) showing diffuse infiltration of mononuclear cells in epicardium (*arrow*) and infiltration of mononuclear cells, degeneration and necrosis of muscle fibers in myocardium (*asterisk*) (100X). Panel **b**: Red muscle section stained with H&E showing infiltration of mononuclear cells, degeneration and necrosis of muscle fibers (*arrow head*) (scale bar = 100 μm). Panel **c**: Liver section showing a blood vessel surrounded by a mononuclear inflammatory cell infiltrate (perivasculitis) focal hepatocellular necrosis (*arrow head*) (scale bar = 100 μm)

**Fig. 3:**
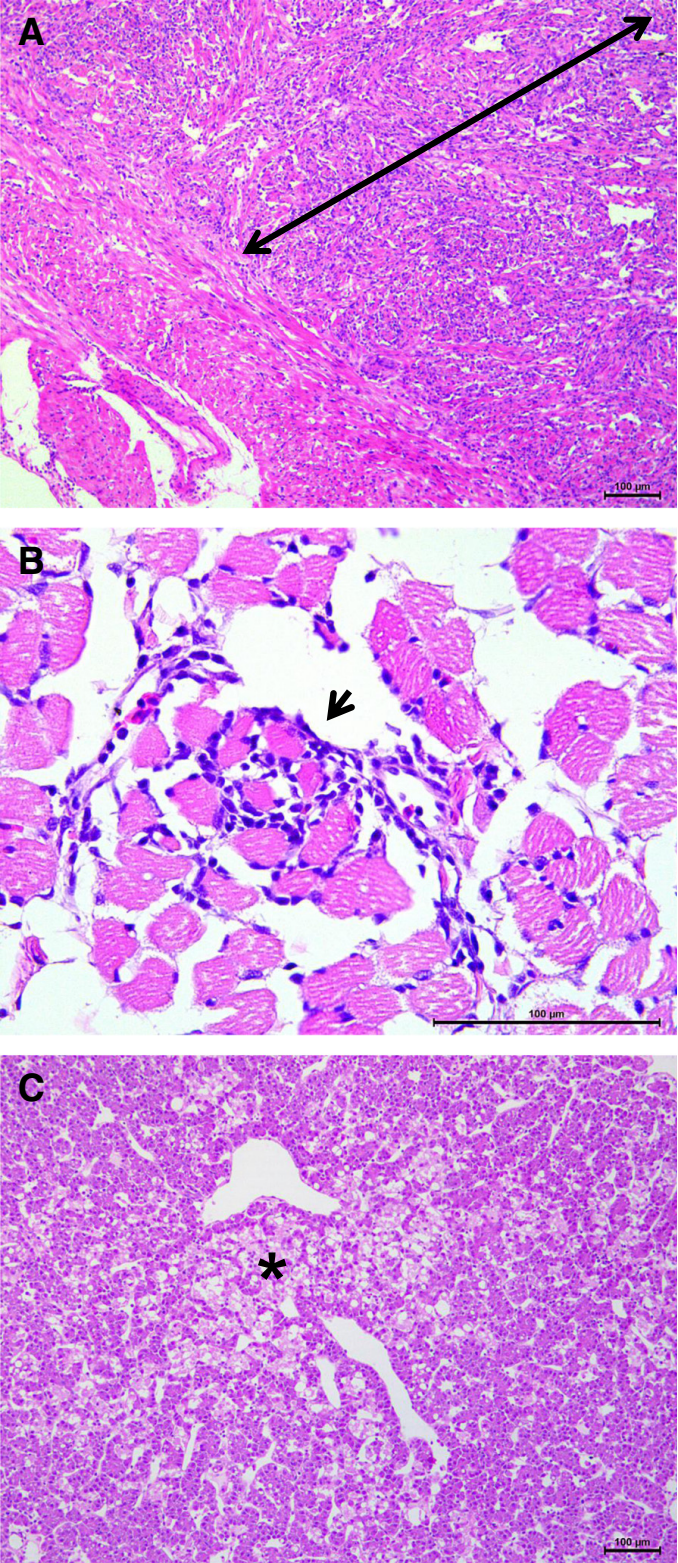
Photomicrographs showing the main microscopic findings in farmed Chilean coho salmon *Oncorhynchus kisutch* with HSMI-like disease. Panel **a**: Heart section stained with haematoxylin and eosin (H&E) showing infiltration of the spongious layer (*arrow*) by mononuclear cells (scale bar = 100 μm). Panel **b**: Red muscle section stained with H&E showing a focal infiltration of mononuclear cells among muscle fiber bundles (*arrow head*) (scale bar = 100 μm). Panel **c** Liver section showing focal necrosis of hepatocytes (*asterisk*) close to a blood vessel (scale bar = 100 μm)

Table 2 summarizes the nature and frequency of the significant histopathology lesions in tissues of Atlantic salmon affected with HSMI and coho salmon affected with HSMI-like disease. The microscopic lesions in Atlantic salmon are shown in Fig. 2, and those in coho salmon are shown in Fig. 3. In Atlantic salmon the affected fish presented with epicarditis and myocarditis characterized by infiltration of mononuclear cells, and degeneration and necrosis in muscle fibers in myocardium (Fig. 2a). The coho salmon had similar lesions in the epicardium but unlike in Atlantic salmon, myocarditis was present generally only in the spongy layer (Fig. 3a). In Atlantic salmon the red muscle had multifocal to diffuse infiltration of mononuclear cells and in some cases with degeneration and necrosis (Fig. 2b). The inflammatory response in red muscle in coho salmon was similar but mild to moderate in severity (Fig. 3b). In Atlantic salmon and coho salmon the most frequent lesion in the liver was blood vessels surrounded by a mononuclear inflammatory cell infiltrate (perivasculitis) and necrosis (Figs. 2c and 3c). The spleen and kidney in both species had mononuclear infiltrations; however nephritis was more frequently seen in coho salmon. The posterior gut in both species had mononuclear cell infiltration in the lamina propria and sub mucosa. The histopathological changes in the gill were more evident in coho salmon, and presented as multifocal to diffuse lamellar fusion with interlamellar vesicle formation associated to *Paramoeba perurans* and epitheliocystis-like epithelial inclusions.

Overall the gross and histopathological lesions in farmed Atlantic salmon in Chile are identical to HSMI lesions described in marine-farmed Atlantic salmon in Norway and Scotland [15, 16], which is the classical presentation of HSMI in farmed Atlantic salmon. The histopathological lesions we describe in coho salmon in the present study are different from those reported in Atlantic salmon [15, 16], and in rainbow trout with the new HSMI-like disease in Norway [27]. In the Chilean coho salmon cases, the heart inflammation is diffuse and present generally in the spongy layer; the red muscle inflammation is mild and not always present (Table 2). Cardiomyopathy (CMS) and Pancreas disease (PD) should be considered in the differential diagnosis. CMS is a viral disease of farmed Atlantic salmon caused by piscine myocarditis virus (PMCV) [21, 28] characterized by epicarditis, massive cell infiltration, degeneration and necrosis of the spongy myocardium and inflammatory infiltration of epicardium [29, 30], but is neither associated with liver necrosis nor myositis like the coho salmon affected by HSMI-like disease in the present study. Moreover, the fish were negative in the RT-qPCR assay for PMCV. The second differential diagnosis, PD, in farmed Atlantic salmon and rainbow trout is caused by salmonid alphavirus (SAV) [27, 31]. The characteristic histopathological findings of PD include cardiomyopathy and skeletal myopathy [32], however, the pancreatic lesions are an important morphological change [27]. All samples analyzed in the present study were negative in the RT-qPCR assay for SAV. Thus, the present study reports coho salmon cases with gross pathology lesions not described previously and a marked inflammation of the spongious layer in the heart and inflammation of the red muscle in the affected fish; both findings not previously described. In order to better describe the disease observed in coho salmon, and further link it to PRV, one would have to reproduce the disease experimentally. However, this is out of the scope of this paper, but would be important for future PRV studies.

### Field variation of susceptibility to PRV by host species

Although PRV has been reported in Norway, Ireland, Chile, Canada, and USA, to date HSMI lesions with presence of PRV have only been described in marine-farmed Atlantic salmon in Norway. This is the first report of HSMI lesions with presence of PRV in farmed Atlantic salmon outside of Europe, and the first detection of HSMI-like disease with presence of PRV in coho salmon in Chile. The clinical presentation in coho salmon included signs associated with Jaundice coho salmon syndrome. A similar jaundice syndrome, commonly referred to as yellow fish, which occurs in British Columbia-Canada, was also associated with PRV in Chinook salmon, but the condition could not be reproduced using kidney and liver tissues from affected fish and injecting them into naïve Chinook salmon, sockeye salmon *O. nerka*, Atlantic salmon, and Pacific herring *Clupea pallasii*, and only minimal viral replication could be demonstrated by RT-qPCR [11]. Most recently in Norway, HSMI-like disease was described in rainbow trout [23]. It is probable that PRV is also infecting rainbow trout in Chile and more work would be needed to establish if the virus is associated with any form of clinical disease. It will therefore be important to include in the surveillance the rainbow trout tissues, and to use the molecular tools used by Olsen et al. [23] for detecting PRV-related virus, because the virus in rainbow trout in Chile may be presenting differently from that in Norway.

### Screening for PRV by RT-qPCR

**Fig. 4:**
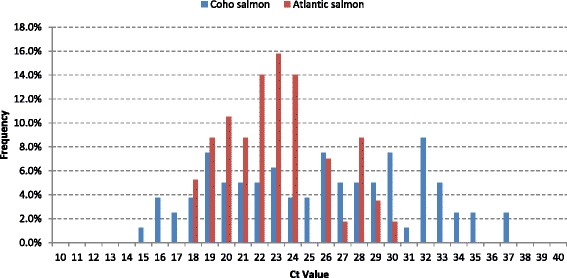
Frequency distribution of the cycle threshold (Ct) values for all farms positive for PRV. The PRV positive Atlantic salmon (*n* = 57) are shown in red, and the PRV positive coho salmon (*n* = 80) are shown in blue

All clinical samples in this study taken for histopathological analysis were also tested for PRV by RT-qPCR assay [33]. The 57 pooled tissue samples from Atlantic salmon from two farm sites and 80 pooled tissue samples from coho salmon from two farm sites were positive for PRV. 36 of the 57 Atlantic salmon tested for PRV and 18 of the 80 coho salmon tested for PRV were subjected to histopathology (Table 2). The distribution of Ct values for Atlantic salmon and coho salmon farms is shown in Fig. 4. The distribution of Ct values in both fish species show several samples with low Ct values (<25) that are indicative of high viral loads, which is consistent with the gross pathology and histopathology findings described above in both cases. The Ct values ranged from 16 to 37 for coho salmon and 18 to 30 for Atlantic salmon. The range was narrower in Atlantic salmon (with a median Ct value of 22.7) compared to coho salmon (median Ct value of 25.75). The data for Atlantic salmon are consistent with the generalized additive logistic regression plot that was described by Løvoll et al. [34] showing the relationship between the probability of samples originating from an HSMI outbreak and PRV RT-qPCR Ct values [35]. It shows that Ct values <25 have a high probability of a sample representing an HSMI outbreak.

### Phylogenetic analysis and sequence diversity of PRV genomic segment S1

**Fig. 5:**
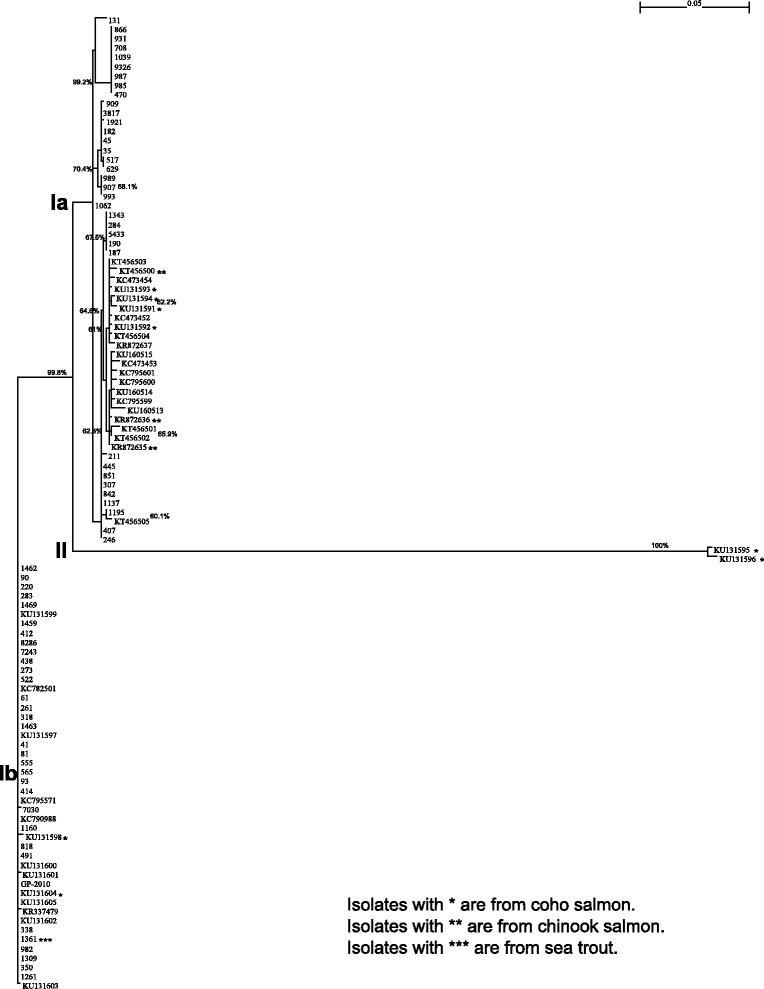
Phylogenetic tree of segment S1 sequences from PRV isolates from Norway, Chile, and Canada. The phylogenetic tree was constructed using the Maximum Likelihood method (software package: PhyML 3.0) [53]. An outgroup (Genbank accession number: AF059720) was used to determine its root, but the outgroup itself was not included in the tree. The bootstrapping procedure was applied for 1000 times and the branches with 60 % or higher bootstrapping support values were marked

All the samples tested yielded the expected PCR product of about 1081 bp. All the PCR products were sequenced and the sequences obtained have been deposited in GenBank [24]; their GenBank Accession numbers are shown in the table in an additional file (see Additional file 2). For each sequence, the sample was sequenced two times at CIBA, once by Macrogen Inc, and once in Canada, and in all four cases of a sample the same sequence was obtained. The table in Additional file 2 shows all the PRV segment S1 nucleotide sequences analysed in this study. Among the Norwegian 180 PRV sequences recently reported by Garseth et al. [5], 60 of them are segment S1 sequences. In the phylogenetic tree prepared by Garseth et al. [5], PRV isolates 131 and 1062 do not belong to any group, indicating that these groups might need to be revised soon. These 60 sequences were merged with the PRV segment S1 sequences from Kibenge et al. [8], some other PRV segment S1 sequences deposited in GenBank [24] by other researchers, and the new Chilean PRV segment S1 sequences obtained in this study (Additional file 2), and a phylogenetic tree was constructed using Maximum Likelihood method, with genome segment S3 of avian reovirus strain 176 (GenBank Accession number AF059720) as the out group sequence [8]. The phylogenetic tree obtained is presented in Fig. 5. The same tree was generated using Neighbor-Joining method (data not shown). Although an outgroup was used to determine the root of the tree, the outgroup itself was not shown in the tree so that we have a detailed graphical display of the tree. In this figure bootstrapping values of more than 60 % are shown. It can be seen that the new Norwegian 60 sequences completely support the sub-genotypes Ia and Ib classification of Kibenge et al. [8]; Garseth et al. [5] Groups II, III, and IV belong to sub-genotype Ia (Norway-Canada subgroup) together with three new Chile PRV cases from coho salmon (GenBank Accession numbers KU131591, KUI31592, KU131593). Moreover, these new Chile PRV cases from coho salmon are most similar to Garseth et al. [5] Group II and the Canadian PRV isolates. Interestingly, a PRV sequence obtained from a market-purchased fish sold in Vancouver, BC, as product from Iceland (GenBank Accession no. KT456505) was distinctly different from Canadian PRV isolates and clustered with Norwegian PRV isolates of sub-genotype Ia (Fig. 5). Garseth et al. [5] Group I (which includes the PRV from sea trout) belongs to sub-genotype Ib (Norway-Chile subgroup) together with the new Chile PRV cases from Atlantic salmon (GenBank Accession numbers KU131597, KUI31599, KU131600, KU131601, KU131603, KU131605) and two new Chile PRV cases from coho salmon (GenBank Accession numbers KU131598, KU131604).

Figure 5 shows that all the Chilean PRV strains from Atlantic salmon grouped as sub-genotype Ib, the Chilean PRV strains from coho salmon were more diversified, grouping in both sub-genotypes Ia and Ib and others forming a distinct new phylogenetic cluster as a separate genotype, Genotype II. In contrast, all the Canadian PRV strains reported to date, regardless of fish species source (i.e., Atlantic salmon, coho salmon and Chinook salmon), group as sub-genotype Ia. The Norwegian PRV-related virus (GenBank Accession no. LN680851) is placed together with the Chilean PRV strains from coho salmon (GenBank Accession numbers KU131595, KU131596) making up the Genotype II cluster. Because the LN680851 sequence is only 561 bp, it distorts the accuracy of the phylogenetic tree (data not shown).

### Genomic segment S1 of PRV Genotype II

It is interesting to note that the full segment S1 sequence (1081 nucleotides) of PRV Genotype II (GenBank Accession numbers KU131595, KU131596) was amplified using the primers and conditions described by Kibenge et al. [8]. The PRV S1 genome segment is bicistronic, encoding the 330-aa Outer clamp (σ3) protein and a 124-aa non-structural protein designated p13 that induces cytotoxicity [36] and may be relevant for virulence of PRV. Therefore the S1 encoded proteins of PRV sub-genotypes Ia and Ib, and Genotype II were compared in detail. Construction of pairwise sequence identity profiles has been used to define genotypes inside species of viruses [28, 37, 38]. Tables 3 and 4 show the percent sequence identities on segment S1 between 23 selected PRV isolates from sub-genotypes Ia and Ib and the three PRV isolates in Genotype II. Both nucleotide and amino acid identity cut-off values of 97.1 % completely correlated with sub-genotypes Ia and Ib and Genotype II in the σ3 gene. In addition, PRV sub-genotype Ib had less genetic variation (nucleotide identity of 99.6–100 %) than sub-genotype Ia (nucleotide identity of 98.0–99.9 %). The two Chilean PRV S1 sequences of Genotype II had 99.5 % nucleotide identity. The nucleotide and amino acid sequence identities between sub-genotypes Ia and Ib strains were both ≤ 96.4 %, whereas between sub-genotype Ib and Genotype II, the nucleotide sequence identity was ≤ 79.2 % and the amino acid sequence identity was ≤ 78.3 %. PRV sub-genotype Ia and Genotype II had nucleotide sequence identity of ≤ 80.9 % and amino acid sequence identity of ≤ 79.0 % (Table 3). Thus Genotype II was more similar to sub-genotype Ia than to Ib in the σ3 gene. In Table 3, sequence homology of Genotype II sequence KU131596 with the most distant sub-genotype Ib PRV sequence (KU131598) at the nucleotide level is 78.7 %, and the amino acid sequence homology of the σ3 protein is 77.2 %. These comparisons did not include the LN680851 sequence, of which only 561 nt or 187 aa of the σ3 gene are available. In a 494 nt overlap and 164 aa overlap, sub-genotype 1a sequence KU160513 with the lowest identities with Genotype II, had 84.6 and 79.9 % nucleotide and amino acid sequence identities, respectively, with LN680851; sub-genotype 1b sequence KU131598 also with the lowest identities with Genotype II, had 83.4 and 79.3 % nucleotide and amino acid sequence identities, respectively, with LN680851; Genotype II sequence KU131596 with the lowest identities with either sub-genotype 1a or Ib, had 97.8 and 98.2 % nucleotide and amino acid sequence identities, respectively, with LN680851. This analysis confirms the phylogenetic analysis (Fig. 5) showing that all the Chilean PRV strains from Atlantic salmon grouped as sub-genotype Ib whereas the Chilean PRV strains from coho salmon grouped in both sub-genotypes Ia and Ib and Genotype II.

**Table 3:** Pairwise sequence comparison of Segment S1 of selected PRV strains showing Genotypes I (sub-genotypes Ia and Ib) and II^1^

Genotype	Ia															Ib								II			
**PRV isolate** ^**2**^	1	**2**	**3**	4	**5**	6	7	8	9	10	11	12	13	14	15	**16**	17	**18**	19	**20**	21	**22**	**23**	**24**	**25**	26	**Ia**
KT456503	-	**99.9**	**99.8**	99.8	**99.6**	99.6	99.6	99.6	99.6	99.5	99.3	99.3	99.2	99.0	98.7	**96.0**	96.0	**96.0**	96.0	**96.0**	96.0	**95.9**	**95.8**	**80.7**	**80.5**	nd	
**KU131593** ^**a**^	**100**	**-**	**99.6**	**99.6**	**99.5**	**99.5**	**99.5**	**99.5**	**99.5**	**99.4**	**99.2**	**99.2**	**99.0**	**98.9**	**98.6**	**95.9**	**95.9**	**95.9**	**95.9**	**95.9**	**95.9**	**95.8**	**95.7**	**80.9**	**80.3**	**nd**	
**KU131594** ^**a**^	**100**	**100**	**-**	**99.5**	**99.6**	**99.4**	**99.4**	**99.4**	**99.4**	**99.3**	**99.0**	**99.0**	**98.9**	**98.9**	**98.4**	**95.8**	**95.8**	**95.8**	**95.8**	**95.8**	**95.8**	**95.7**	**95.5**	**80.6**	**80.4**	**nd**	
KC473454	99.3	**99.3**	**99.3**	-	**99.4**	99.4	99.4	99.4	99.4	99.3	99.0	99.0	98.9	98.8	98.4	**95.8**	95.8	**95.8**	95.8	**95.8**	95.8	**95.7**	**95.5**	**80.5**	**80.3**	**nd**	
**KU131591** ^**a**^	**100**	**100**	**100**	**99.3**	**-**	**99.3**	**99.3**	**99.3**	**99.3**	**99.2**	**98.9**	**98.9**	**98.8**	**98.8**	**98.3**	**95.7**	**95.7**	**95.7**	**95.7**	**95.7**	**95.7**	**95.5**	**95.4**	**80.7**	**80.5**	**nd**	
KR872636	99.6	**99.6**	**99.6**	98.9	**99.6**	-	99.3	99.5	99.5	99.6	99.4	99.4	99.0	99.2	98.6	**95.9**	95.9	**95.9**	95.9	**95.9**	95.9	**95.8**	**95.7**	**80.6**	**80.4**	nd	
KT456500^b^	98.9	**98.9**	**98.9**	98.2	**98.9**	98.6	-	99.3	99.3	99.2	98.9	99.2	98.8	98.7	98.3	**95.7**	95.7	**95.7**	95.7	**95.7**	95.7	**95.5**	**95.4**	**80.4**	**80.2**	nd	
JN991006	100	**100**	**100**	99.3	**100**	99.6	98.9	-	99.8	99.4	99.2	99.2	99.3	98.9	98.8	**96.3**	96.3	**96.3**	96.3	**96.3**	96.3	**96.1**	**96.0**	**80.6**	**80.4**	nd	
HG329868	100	**100**	**100**	99.3	**100**	99.6	98.9	100	-	99.4	99.2	99.2	99.5	98.9	99.0	**96.4**	96.4	**96.4**	96.4	**96.4**	96.4	**96.3**	**96.1**	**80.9**	**80.6**	nd	
KU160514	99.6	**99.6**	**99.6**	98.9	**99.6**	99.3	98.6	99.6	99.6	-	99.3	99.5	98.9	99.3	98.4	**95.8**	95.8	**95.8**	95.8	**95.8**	95.8	**95.7**	**95.5**	**80.2**	**80.3**	nd	
KT456501	98.6	**98.6**	**98.6**	97.8	**98.6**	98.2	97.5	98.6	98.6	98.2	-	99.0	98.7	98.9	98.2	**95.5**	95.5	**95.5**	95.5	**95.5**	95.5	**95.4**	**95.3**	**80.5**	**80.3**	nd	
KC473453	99.6	**99.6**	**99.6**	98.9	**99.6**	99.3	99.3	99.6	99.6	99.3	99.3	-	98.7	99.0	98.2	**95.5**	95.5	**95.5**	95.5	**95.5**	95.5	**95.4**	**95.3**	**80.3**	**80.0**	nd	
KT456505	98.6	**98.6**	**98.6**	97.8	**98.6**	98.2	97.5	98.6	98.6	98.2	97.1	98.2	-	98.4	98.6	**96.1**	96.1	**96.1**	96.1	**96.1**	96.1	**96.0**	**95.9**	**80.9**	**80.7**	nd	
KU160513	98.9	**98.9**	**98.9**	98.2	**98.9**	98.6	97.8	98.9	98.9	98.6	97.5	98.6	97.5	-	98.0	**95.3**	95.3	**95.3**	95.3	**95.3**	95.3	**95.2**	**95.1**	**80.2**	**79.9**	nd	
HG329848	100	**100**	**100**	99.3	**100**	99.6	98.9	100	100	99.6	98.6	99.6	98.6	98.9	-	**96.4**	96.4	**96.4**	96.4	**96.4**	96.4	**96.3**	**96.1**	**80.9**	**80.7**	nd	
**KU131597**	**96.4**	**96.4**	**96.4**	**95.7**	**96.4**	**96.0**	**95.3**	**96.4**	**96.4**	**96.0**	**95.3**	**96.0**	**95.3**	**95.3**	**96.4**	**-**	**100**	**100**	**100**	**100**	**100**	**99.9**	**99.8**	**79.2**	**79.0**	**nd**	**Ib**
HG329897^c^	96.4	**96.4**	**96.4**	95.7	**96.4**	96.0	95.3	96.4	96.4	96.0	95.3	96.0	95.3	95.3	96.4	**100**	-	**100**	100	**100**	100	**99.9**	**99.8**	**79.2**	**79.0**	nd	
**KU131602**	**96.4**	**96.4**	**96.4**	**95.7**	**96.4**	**96.0**	**95.3**	**96.4**	**96.4**	**96.0**	**95.3**	**96.0**	**95.3**	**95.3**	**96.4**	**100**	**100**	**-**	**100**	**100**	**100**	**99.9**	**99.8**	**79.2**	**79.0**	**nd**	
KC795571	96.4	**96.4**	**96.4**	95.7	**96.4**	96.0	95.3	96.4	96.4	96.0	95.3	96.0	95.3	95.3	96.4	**100**	100	**100**	-	**100**	100	**99.9**	**99.8**	**79.2**	**79.0**	nd	
**KU131604** ^**a**^	**96.4**	**96.4**	**96.4**	**95.7**	**96.4**	**96.0**	**95.3**	**96.4**	**96.4**	**96.0**	**95.3**	**96.0**	**95.3**	**95.3**	**96.4**	**100**	**100**	**100**	**100**	**-**	**100**	**99.9**	**99.8**	**79.2**	**79.0**	**nd**	
GU994022	96.4	**96.4**	**96.4**	95.7	**96.4**	96.0	95.3	96.4	96.4	96.0	95.3	96.0	95.3	95.3	96.4	**100**	100	**100**	100	**100**	-	**99.9**	**99.8**	**79.2**	**79.0**	nd	
**KU131603**	**96.0**	**96.0**	**96.0**	**95.3**	**96.0**	**95.7**	**94.9**	**96.0**	**96.0**	**95.7**	**94.9**	**95.7**	**94.9**	**94.9**	**96.0**	**99.6**	**99.6**	**99.6**	**99.6**	**99.6**	**99.6**	**-**	**99.6**	**79.1**	**78.8**	**nd**	
**KU131598** ^**a**^	**95.7**	**95.7**	**95.7**	**94.9**	**95.7**	**95.3**	**94.6**	**95.7**	**95.7**	**95.3**	**94.6**	**95.3**	**94.6**	**94.6**	**95.7**	**99.3**	**99.3**	**99.3**	**99.3**	**99.3**	**99.3**	**98.9**	**-**	**79.0**	**78.7**	**nd**	
**KU131595** ^**a**^	**79.0**	**79.0**	**79.0**	**78.3**	**79.0**	**79.0**	**78.3**	**79.0**	**79.0**	**79.0**	**78.3**	**79.0**	**79.0**	**78.3**	**79.0**	**78.3**	**78.3**	**78.3**	**78.3**	**78.3**	**78.3**	**77.9**	**77.5**	**-**	**99.5**	**nd**	**II**
**KU131596** ^**a**^	**78.6**	**78.6**	**78.6**	**77.9**	**78.6**	**78.6**	**77.9**	**78.6**	**78.6**	**78.6**	**77.9**	**78.6**	**78.6**	**77.9**	**78.6**	**77.9**	**77.9**	**77.9**	**77.9**	**77.9**	**77.9**	**77.5**	**77.2**	**99.3**	**-**	**nd**	
LN680851^d^	nd	**nd**	**Nd**	nd	**nd**	Nd	nd	nd	nd	Nd	nd	nd	nd	nd	nd	**nd**	nd	**nd**	nd	**nd**	nd	**nd**	**nd**	**nd**	**nd**	-	

^1^Pairwise sequence comparision was done with EMBOSS Water on-line program using default settings [52]; values above the diagonal are nucleotide sequence identities (%) in 830 nt overlap; values below the diagonal are deduced amino acid sequence identities of Outer clamp (σ3) protein (%) in 276 aa overlap. Bold text denotes new Chilean PRV sequences generated in this study

^2^PRV isolates identified in vertical column by GenBank Accession Numbers and on the top horizontal row by order of listing in the vertical column

^3^nd denotes not done because LN680851 available sequence is only 561 nt or 187 aa. ^a^Denotes PRV “isolates” from coho salmon; ^b^Denotes PRV “isolate” from Chinook salmon; ^c^Denotes PRV “isolate” from sea trout. ^d^Denotes PRV “isolate” from rainbow trout

**Table 4:** Pairwise comparison of amino acid sequence of p13 protein of selected PRV strains showing Genotypes I (sub-genotypes Ia and Ib) and II^1^

Genotype	Ia															Ib								II			
**PRV isolate** ^**2**^	1	**2**	**3**	4	**5**	6	7	8	9	10	11	12	13	14	15	**16**	17	**18**	19	20	21	**22**	**23**	**24**	**25**	26	**Ia**
KT456503	-																										
**KU131593** ^**a**^	**99.2**	**-**																									
**KU131594** ^**a**^	**100**	**99.2**	**-**																								
KC473454	100	**99.2**	**100**	-																							
**KU131591** ^**a**^	**98.4**	**97.6**	**98.4**	**96.8**	**-**																						
KR872636	98.4	**97.6**	**98.4**	96.8	**96.8**	-																					
KT456500^b^	100	**99.2**	**100**	98.4	**98.4**	98.4	-																				
JN991006	98.4	**97.6**	**98.4**	96.8	**98.4**	98.4	98.4	-																			
HG329868	99.2	**98.4**	**99.2**	97.6	**99.2**	99.2	99.2	99.2	-																		
KU160514	97.6	**96.8**	**97.6**	96.0	**99.2**	99.2	97.6	97.6	98.4	-																	
KT456501	96.8	**96.0**	**96.8**	95.2	**98.4**	98.4	96.8	96.8	97.6	97.6	-																
KC473453	97.6	**96.8**	**97.6**	96.0	**99.2**	99.2	97.6	97.6	98.4	100	97.6	-															
KT456505	97.6	**96.8**	**97.6**	96.0	**97.6**	97.6	97.6	97.6	98.4	96.8	96.0	96.8	-														
KU160513	96.8	**96.0**	**96.8**	95.2	**98.4**	98.4	96.8	96.8	97.6	99.2	96.8	99.2	96.0	-													
HG329848	99.2	**98.4**	**99.2**	97.6	**99.2**	99.2	99.2	99.2	100	98.4	97.6	98.4	98.4	97.6	-												
**KU131597**	**92.7**	**91.9**	**92.7**	**91.9**	**92.7**	**92.7**	**92.7**	**93.5**	**93.5**	**91.9**	**91.1**	**91.9**	**91.9**	**91.1**	**93.5**	**-**											**Ib**
HG329897^c^	92.7	**91.9**	**92.7**	91.9	**92.7**	92.7	92.7	93.5	93.5	91.9	91.1	91.9	91.9	91.1	93.5	**100**	-										
**KU131602**	**92.7**	**91.9**	**92.7**	**91.9**	**92.7**	**92.7**	**92.7**	**93.5**	**93.5**	**91.9**	**91.1**	**91.9**	**91.9**	**91.1**	**93.5**	**100**	**100**	**-**									
KC795571	92.7	**91.9**	**92.7**	91.9	**92.7**	92.7	92.7	93.5	93.5	91.9	91.1	91.9	91.9	91.1	93.5	**100**	100	**100**	-								
**KU131604** ^**a**^	**92.7**	**91.9**	**92.7**	**91.9**	**92.7**	**92.7**	**92.7**	**93.5**	**93.5**	**91.9**	**91.1**	**91.9**	**91.9**	**91.1**	**93.5**	**100**	**100**	**100**	**100**	**-**							
GU994022	92.7	**91.9**	**92.7**	91.9	**92.7**	92.7	92.7	93.5	93.5	91.9	91.1	91.9	91.9	91.1	93.5	**100**	100	**100**	100	100	-						
**KU131603**	**92.7**	**91.9**	**92.7**	**91.9**	**92.7**	**92.7**	**92.7**	**93.5**	**93.5**	**91.9**	**91.1**	**91.9**	**91.9**	**91.1**	**93.5**	**100**	**100**	**100**	**100**	**100**	**100**	**-**					
**KU131598** ^**a**^	**91.9**	**91.1**	**91.9**	**91.9**	**91.1**	**91.9**	**91.9**	**92.7**	**92.7**	**91.1**	**90.3**	**91.1**	**91.1**	**90.3**	**92.7**	**99.2**	**99.2**	**99.2**	**99.2**	**99.2**	**99.2**	**99.2**	**-**				
**KU131595** ^**a**^	**79.8**	**80.6**	**79.8**	**80.6**	**79.8**	**79.8**	**79.8**	**79.8**	**80.6**	**79.0**	**78.2**	**79.0**	**79.8**	**78.2**	**80.6**	**78.2**	**78.2**	**78.2**	**78.2**	**78.2**	**78.2**	**78.2**	**77.4**	**-**			**II**
**KU131596** ^**a**^	**79.8**	**80.6**	**79.8**	**79.8**	**80.6**	**79.8**	**79.8**	**79.8**	**80.6**	**79.0**	**78.2**	**79.0**	**79.8**	**78.2**	**80.6**	**78.2**	**78.2**	**78.2**	**78.2**	**78.2**	**78.2**	**78.2**	**77.4**	**100**	**-**		
LN680851^d^	79.8	**80.6**	**79.8**	79.8	**80.6**	79.8	79.8	79.8	80.6	79.0	78.2	79.0	80.6	78.2	78.2	**78.2**	78.2	**78.2**	78.2	78.2	78.2	**78.2**	**77.4**	**97.6**	**97.6**	-	

^1^Pairwise sequence comparision was done with EMBOSS Water on-line program [52]. Bold text denotes new Chilean PRV sequences generated in this study

^2^PRV isolates identified in vertical column by GenBank Accession Numbers and on the top horizontal by order of listing in vertical column

^a^Denotes PRV “isolates” from coho salmon; ^b^Denotes PRV “isolate” from Chinook salmon; ^c^Denotes PRV “isolate” from sea trout. ^d^Denotes PRV “isolate” from rainbow trout

The pairwise amino acid sequence analysis of p13 protein is shown in Table 4. The sequence identity within sub-genotype Ia strains was ≤ 96.0 %; within Ib and Genotype II strains were ≤ 99.2 and ≤ 97.6 %, respectively. The sequence identity between sub-genotypes Ia and Ib strains was ≤ 93.5 %, whereas between sub-genotypes Ia and Genotype II it was ≤ 80.6 %, and between sub-genotypes Ib and Genotype II it was ≤ 78.2 %, Interestingly, some sub-genotypes Ia strains also had 78.2 % amino acid identity with Genotype II strains suggesting that there is no amino acid identity cut-off value between sub-genotypes Ia and Ib and Genotype II for the p13 protein.

### Phylogenetically PRV sub-genotype Ib appears to be the original PRV genotype

Figure 5 suggests that PRV sub-genotype Ib was the original virus that gave rise to sub-genotype Ia and Genotype II later. Although the geographical areas the PRV sub-genotypes Ia and Ib covered in Norway and Chile are overlapping (even in the same fish farms), Ib and Ia appear to spread independently as if they were two different viruses. It is interesting to note that to date we and others [11, 12, 14] have not found Genotype II sequence in any fish species in Canada but this sequence (KU131596 with the lowest identities with either sub-genotype 1a or Ib) had 97.8 and 98.2 % nucleotide and amino acid sequence identities, respectively, with the Norwegian PRV-related virus (GenBank Accession no. LN680851) (Tables 3 and 4). The absence of Genotype II sequence in Canada is not related to differences in RT-qPCR methods used to screen for PRV (for example Haugland et al. [33] versus Løvoll et al. [21]) since we were able to detect it in Chilean farmed coho salmon and to amplify the full segment S1 sequence using the same primers and conditions as we use in Canada [8].

## Conclusions

To our knowledge the present work constitutes the first published report of HSMI lesions with presence of PRV in farmed Atlantic salmon outside of Europe, and the first report of HSMI-like lesions with presence of PRV in coho salmon in Chile. The Chilean PRV strains from coho salmon are more genetically diversified than those from Atlantic salmon, and some form a distinct new phylogenetic cluster, designated Genotype II.

## Methods

### Field sampling

For the clinical and histopathological description of HSMI, the farm sites were chosen to fulfill the following characteristics in order to increase the probability of detecting HSMI disease (with the goal to establish a causal relationship between PRV and HSMI): targeted sampling at peak mortality during the disease outbreak (i.e., at the height of clinical expression), HSMI clinical disease in the absence of any pathogen other than PRV, and the samples for histopathological examination were taken from selected fish showing HSMI gross pathology. The targeted sampling on the farm was made by a senior Veterinarian with experience in recognizing the enzootic clinical conditions [infectious pancreatic necrosis virus (IPNV), salmonid rickettsial septicaemia (SRS), bacterial kidney disease (BKD), furunculosis, etc.] and HSMI. The general epidemiological data (location, mortality, other diseases, fish size) were collected. The Atlantic salmon farm sites were located in XI region, and the coho salmon farm sites were located in X Region, Chile. For Atlantic salmon, average weight and cumulative mortality data were not available for cases in sea water. The freshwater cases had average weight of 30 to 60 g and cumulative mortality ranging from 18 to 53 % and had mixed BKD and fungal infections. The coho salmon cases were all in sea water and had an average weight of 721 or 1594 g, cumulative mortality of 2.3 % or 4.6 %, with fracture of the column, SRS, and icterus. Whole fish (65 Atlantic salmon and 85 coho salmon) were transported on ice to ETECMA, CIBA Laboratory, for pathological analysis.

### Field sampling for PRV testing by RT-qPCR and genetic characterization of PRV

In total, two Atlantic salmon and two coho salmon farm sites were studied. Individual and pooled tissue samples (heart, kidney, and liver) were collected directly in ethanol 70 % (v/v) or RNAlater® (Ambion Inc) and were delivered to ETECMA, CIBA Laboratory, for analysis. 57 pooled tissue samples from Atlantic salmon from two farm sites and 80 pooled tissue samples from coho salmon from two farm sites were screened for PRV by RT-qPCR and the cycle threshold (Ct) values were plotted and statistically analyzed.

### Necropsy and histopathology

Post mortem was performed on whole fish carcasses. The significant external and internal gross lesions were registered and the frequency of each of these was determined and plotted. The gill, heart, liver, spleen, kidney, skeletal muscle, pyloric caeca and gut of 36 Atlantic salmon specimens from two farms corresponding to four sampling times, and 18 coho salmon specimens from two farms, corresponding to four sampling times, were examined histologically. Tissue samples for histological analysis were fixed in 10 % neutral buffered formalin. They were then processed using standard procedures and the sections, 3–4 μm, were stained by haematoxylin & eosin (H&E) according to Prophet et al. [39], in order to describe the significant microscopic morphological changes.

### Real time RT-PCR (RT-qPCR)

For the cases investigated, an automated tissue homogenization of samples was performed using the MagNA Lyser instrument (Roche). Total RNA was extracted using a robot (Roche MagNA Pure LC instrument) with the MagNA Pure LC RNA isolation kit III - Tissue (for virus) and Isolation kit III Tissue (for bacteria and fungi), according to the manufacturer's instructions. In both cases, the extracted RNA was eluted in 50 μl of nuclease-free water, and RNA yields were quantified and purity analysed using the OD260/280 ratio and a NanoPhotometer® P 300 (Implen). The eluted RNA was tested immediately following quantitation. The RT-qPCR analysis was made using Light-Cycler 480 RNA Master Hydrolysis Probes for RNA (Roche). The RT-qPCR was done using PRV specific primers and conditions as described by Haugland et al. [33]. Samples were considered PRV positive based on Ct values according to the laboratory validated procedure. To ensure efficient performance of each assay, a constitutively expressed endogenous gene, eukaryotic elongation factor 1-alpha (ELF1α), was used as an internal control reference gene [40]. The cut off Ct for PRV positive samples was 37.6, and for sequence analysis was Ct ≤ 30 for PRV and Ct ≤ 20 for ELF1α for all samples.

Fish tissue samples were also tested for viral hemorrhagic septicemia virus (VHSV) using methods described by Garver et al. [41], epizootic hematopoietic necrosis virus (EHNV) and infectious hematopoietic necrosis virus (IHNV) using methods described by Holopainen et al. [42], salmon alphavirus (SAV) using methods described by Hodneland et al. [43], infectious salmon anaemia virus (ISAV) by Snow et al. [40], piscine myocarditis virus (PMCV) by Løvoll et al. [21], IPNV by Orpetveit et al. [44], and *P. salmonis* using methods described by Corbeil et al. [45], *Renibacterium salmoninarum* [46] and *Flavobacterium psychrophilum* [47] with minor modifications. Kidney and spleen samples were also cultured on blood agar and tryptic salt agar (TSA) agar for bacterial isolation [48].

### DNA Sequencing and phylogenetic analysis

For DNA sequencing, we included samples that were positive to PRV with Ct value ≤ 30 and ELF1α Ct value ≤ 20. The full length segment S1 of PRV was amplified using the primers and conditions described by Kibenge et al. [8]. Conventional RT-PCR was carried out using the EXPRESS qPCR SuperMix® with Premixed ROX® (Invitrogen), in a SwiftTM MaxPro Thermal Cycler® (Esco Healthcare Pte. Ltd.). The PCR products were then purified by agarose gel electrophoresis. Sequencing was performed using the ABI 310 instrument in ETECMA, CIBA Laboratory, using the BigDye Terminator V. 3.1 kit according to the laboratory procedure. Duplicate samples were sequenced commercially by Macrogen Inc. (South Korea). Duplicate total RNA samples were precipitated in 100 % ethanol and submitted to the Virology Research Laboratory (University Prince Edward Island, Canada) for confirmation of the sequencing results.

The sequences were analyzed with Geneious v6.0.4 software [49] and subjected to a BLAST search using programs available via the National Center for Biotechnology Information [50] against the latest release at GenBank [24]. Analysis to identify putative ORFs and their predicted amino acid sequences and other protein characteristics was conducted using the Sequence Manipulation suite, version 2 [51]. Pairwise sequence comparision was done with EMBOSS Water on-line program [52], which uses the Smith-Waterman algorithm (modified for speed enhancements) to calculate the local alignment of two sequences. The evolutionary distance of PRV-S1 gene sequenced in this study, together with some other PRV-S1 sequences accessible in GenBank [24] was estimated using Maximum Likelihood method (software package: PhyML 3.0) [53] and Neighbor-Joining phylogenetic analysis [54]. A bootstrap analysis to investigate the stability of the trees was performed on 1000 replicates (only the bootstrapping values that are greater than 60 % are marked).

### Statistical analysis

The Ct values of all pooled tissue samples were plotted and the median, maximum and minimum were determined for each fish species.

## Additional files

Additional file 1: Movie S1. A field clinical case of heart and skeletal muscle inflammation (HSMI) in Atlantic salmon *Salmo salar* cultivated in seawater cage. The affected fish are lethargic and are swimming near the cage net and facing the sea current. (MP4 341036 kb) Additional file 2: Table S1. Piscine reovirus segment S1 nucleotide sequences analysed in this study [5, 8, 11, 23, 33, 55, 56]. (DOC 176 kb)
