# Supplementary material for: First description of clinical presentation of piscine orthoreovirus (PRV) infections in salmonid aquaculture in Chile and identification of a second genotype (Genotype II) of PRV
Source: Virol J. 2016 Jun 13;13:98. doi: 10.1186/s12985-016-0554-y (PMC4906990; doi:10.1186/s12985-016-0554-y)
Supplement: Additional file 2: Table S1. — Piscine reovirus segment S1 nucleotide sequences analysed in this study [5, 8, 11, 23, 33, 55, 56]. (DOC 176 kb) [file 12985_2016_554_MOESM2_ESM.doc]

**Additional file 2: Table S1.** Piscine orthoreovirus segment S1 nucleotide sequences analyzed in this study

| **PRV isolate ID** | **Country** | **GenBank Accession number** | **Reference** | **Year** | **Genetic group^1^** |
| --- | --- | --- | --- | --- | --- |
| VT02292012-163 | Canada | KC473452 | [8] | 2012 | Ia |
| VT06062012-358 | Canada | KC473453 | [8] | 2012 | Ia |
| VT06202012-371 | Canada | KC473454 | [8] | 2012 | Ia |
| VT02292012-167 | Canada | KC795599 | [8] | 2012 | Ia |
| VT03202012-196 | Canada | KC795600 | [8] | 2012 | Ia |
| VT03202012-209 | Canada | KC795601 | [8] | 2012 | Ia |
| VT09122012-755 | Canada | KT456500 | [55] | 2011 | Ia |
| VT09192013-402 | Canada | KT456501 | [55] | 2013 | Ia |
| VT09192013-408 | Canada | KT456502 | [55] | 2013 | Ia |
| VT01212014-03 | Canada | KT456503 | [55] | 2014 | Ia |
| VT01212014-04 | Canada | KT456504 | [55] | 2014 | Ia |
| VT01212014-09 | Iceland | KT456505 | [55] | 2014 | Ia |
| VT01292015-09 | Canada | KU160513 | [55] | 2015 | Ia |
| VT07222015-106 | Canada | KU160514 | [55] | 2015 | Ia |
| VT07222015-107 | Canada | KU160515 | [55] | 2015 | Ia |
| BCinoc3 | Canada | KR872635 | [11] | 2012 |  |
| BCinoc12_13 | Canada | KR872636 | [11] | 2013 |  |
| BC361_14 | Canada | KR872637 | [11] | 2014 |  |
| 5433-S3 | Norway | JN991006 | [34] | 2012 | Ia |
| 1921-S3 | Norway | JN991007 | [34] | 2012 | Ia |
| 9326-S3 | Norway | JN991008 | [34] | 2012 | Ia |
| 3817-S3 | Norway | JN991012 | [34] | 2012 | Ia |
| 35 Bjoreio | Norway | HG329842 | [5] | 2009 | IV |
| 45 Eira | Norway | HG329843 | [5] | 2009 | IV |
| 131 Gaula | Norway | HG329848 | [5] | 2009 | unknown |
| 182 Hestdal | Norway | HG329849 | [5] | 2009 | IV |
| 187 Hestdal | Norway | HG329850 | [5] | 2009 | II |
| 190 Hestdal | Norway | HG329851 | [5] | 2009 | II |
| 211 Mandal | Norway | HG329852 | [5] | 2009 | II |
| 246 Mandal | Norway | HG329854 | [5] | 2009 | II |
| 284 Stjordal | Norway | HG329858 | [5] | 2009 | II |
| 307 Stjordal | Norway | HG329859 | [5] | 2009 | II |
| 407 Vosso | Norway | HG329863 | [5] | 2009 | II |
| 445 Alta | Norway | HG329868 | [5] | 2008 | II |
| 470 Drevja | Norway | HG329869 | [5] | 2008 | III |
| 517 Eira | Norway | HG329871 | [5] | 2008 | IV |
| 629 Jolstra | Norway | HG329875 | [5] | 2008 | IV |
| 708 Nausta | Norway | HG329876 | [5] | 2008 | III |
| 842 Vikja | Norway | HG329878 | [5] | 2008 | II |
| 851 Vikja | Norway | HG329879 | [5] | 2008 | II |
| 866 Vikja | Norway | HG329880 | [5] | 2008 | III |
| 907 Vosso | Norway | HG329881 | [5] | 2008 | IV |
| 909 Vosso | Norway | HG329882 | [5] | 2008 | IV |
| 931 Alta | Norway | HG329883 | [5] | 2007 | III |
| 985 Ekso | Norway | HG329885 | [5] | 2007 | III |
| 987 Ekso | Norway | HG329886 | [5] | 2007 | III |
| 989 Ekso | Norway | HG329887 | [5] | 2007 | IV |
| 993 Ekso | Norway | HG329888 | [5] | 2007 | IV |
| 1039 Laerdal | Norway | HG329889 | [5] | 2007 | III |
| 1062 Mandal | Norway | HG329890 | [5] | 2007 | unknown |
| 1137 Stjordal | Norway | HG329891 | [5] | 2007 | II |
| 1195 Aaroy | Norway | HG329893 | [5] | 2007 | II |
| 1343 Moelv | Norway | HG329896 | [5] | 2008 | II |
| VT12202013_CGA_2013_4 | Chile | KU131591 | This study | 2013 |  |
| VT12202013_CGA_2013_1 | Chile | KU131592 | This study | 2013 |  |
| VT12202013_CGA_2013_2 | Chile | KU131593 | This study | 2013 |  |
| 2015_CGA_2015_B | Chile | KU131594 | This study | 2015 |  |
| VT12202013_CGA_2013_3 | Chile | KU131595 | This study | 2013 |  |
| VT12202013_CGA_2013_5 | Chile | KU131596 | This study | 2013 |  |
| F445-2013 | Norway | LN680851 | [23] | 2013 |  |
| 61 Eira | Norway | HG329844 | [5] | 2009 | I |
| 81 Etne | Norway | HG329845 | [5] | 2009 | I |
| 90 Etne | Norway | HG329846 | [5] | 2009 | I |
| 93 Etne | Norway | HG329847 | [5] | 2009 | I |
| 220 Mandal | Norway | HG329853 | [5] | 2009 | I |
| 261 Nidelv | Norway | HG329855 | [5] | 2009 | I |
| 273 Skibotn | Norway | HG329856 | [5] | 2009 | I |
| 283 Skjomen | Norway | HG329857 | [5] | 2009 | I |
| 318 Storelva Holt | Norway | HG329860 | [5] | 2009 | I |
| 338 Surna | Norway | HG329861 | [5] | 2009 | I |
| 350 Surna | Norway | HG329862 | [5] | 2009 | I |
| 411 Vosso | Norway | HG329864 | [5] | 2009 | I |
| 412 Vosso | Norway | HG329865 | [5] | 2009 | I |
| 414 Vosso | Norway | HG329866 | [5] | 2009 | I |
| 438 Alta | Norway | HG329867 | [5] | 2008 | I |
| 491 Eira | Norway | HG329870 | [5] | 2008 | I |
| 522 Ekso | Norway | HG329872 | [5] | 2008 | I |
| 555 Fusta | Norway | HG329873 | [5] | 2008 | I |
| 565 Gaula | Norway | HG329874 | [5] | 2008 | I |
| 818 Surna | Norway | HG329877 | [5] | 2008 | I |
| 982 Eira | Norway | HG329884 | [5] | 2007 | I |
| 1160 Surna | Norway | HG329892 | [5] | 2007 | I |
| 1261 Halsan | Norway | HG329894 | [5] | 2009 | I |
| 1309 Eidsdal | Norway | HG329895 | [5] | 2008 | I |
| 1361 Moelv | Norway | HG329897 | [5] | 2008 | I |
| 1459 Etne | Norway | HG329898 | [5] | 2010 | I |
| 1462 Etne | Norway | HG329899 | [5] | 2010 | I |
| 1463 Etne | Norway | HG329900 | [5] | 2010 | I |
| 1469 Etne | Norway | HG329901 | [5] | 2010 | I |
| 7243-S3 | Norway | JN991009 | [34] | 2012 | Ib |
| 7030-S3 | Norway | JN991010 | [34] | 2012 | Ib |
| 8286-S3 | Norway | JN991011 | [34] | 2012 | Ib |
| Salmo/GP-2010/NOR | Norway | GU994022 | [2] | 2010 | Ib |
| 050607 | Norway | KR337479 | [56] | 2007 | Ib |
| CGA337 | Chile | KC782501 | [8] | 2012 | Ib |
| CGA8857 | Chile | KC790988 | [8] | 2012 | Ib |
| CGA280-05 | Chile | KC795571 | [8] | 2012 | Ib |
| 2013_CGA_2013_A | Chile | KU131597 | This study | 2013 |  |
| 2013_CGA_2013_C | Chile | KU131598 | This study | 2013 |  |
| VT02182014_CGA_2013_8 | Chile | KU131599 | This study | 2013 |  |
| VT02182014_CGA_2013_9 | Chile | KU131600 | This study | 2013 |  |
| VT02182014_CGA_2013_10 | Chile | KU131601 | This study | 2013 |  |
| VT02182014_CGA_2013_6 | Chile | KU131602 | This study | 2013 |  |
| VT02182014_CGA_2013_7 | Chile | KU131603 | This study | 2013 |  |
| 2015_CGA_2015_A | Chile | KU131604 | This study | 2015 |  |
| 2015_CGA_2015_C | Chile | KU131605 | This study | 2015 |  |

^1^Genetic group assigned by Kibenge *et al.* [8] or Garseth *et al.* [5].
